# Prognostic and clinicopathological value of m6A regulators in human cancers: a meta-analysis

**DOI:** 10.18632/aging.204371

**Published:** 2022-11-07

**Authors:** Zhangci Su, Leyao Xu, Xinning Dai, Mengyao Zhu, Xiaodan Chen, Yuanyuan Li, Jie Li, Ruihan Ge, Bin Cheng, Yun Wang

**Affiliations:** 1 Hospital of Stomatology, Sun Yat-sen University, Guangzhou, P.R. China; 2 Guanghua School of Stomatology, Sun Yat-sen University, Guangzhou, P.R. China; 3 Guangdong Provincial Key Laboratory of Stomatology, Guangzhou, P.R. China; 4 Department of Orthodontics, School of Stomatology, Capital Medical University, Beijing, P.R. China

**Keywords:** m6A regulators, cancers, prognosis, clinicopathology, meta-analysis

## Abstract

Background: N6-methyladenosine (m6A) is the most abundant epigenetic modification. Although the dysregulation of m6A regulators has been associated with cancer progression in several studies, its relationship with cancer prognosis and clinicopathology is still controversial. Therefore, we evaluated the prognostic and clinicopathological value of m6A regulators in cancers by performing a comprehensive meta-analysis.

Methods: The PubMed, Cochrane Library, Web of Science, and Embase databases were searched up to April 2022. Hazard ratios were used to analyze the association between m6A with prognosis. We also analyze the relationship between m6A and clinicopathology using odds ratios.

Results: METTL3 overexpression predicted poor overall survival and disease-free survival in cancer patients (*p* < 0.001) such as gastric cancer (*p* < 0.001), esophageal squamous cell carcinoma (*p* < 0.001), oral squamous cell carcinoma (*p* = 0.002) and so on. Additionally, METTL3 overexpression was associated with poor pT stage (*p* < 0.001), pN stage (*p* < 0.001), TNM stage (*p* < 0.001), tumor size >5 cm (*p* < 0.001) and vascular invasion (*p* = 0.024). Conversely, METTL14 overexpression was positively associated with better OS (*p* < 0.001), negatively with poor pT stage (*p* = 0.001), pM stage (*p* = 0.002), pN stage (*p* = 0.011) and TNM stage (*p* < 0.001). Moreover, KIAA1429 overexpression was associated with poor OS (*p* = 0.001). YTHDF1 overexpression was also associated with advanced pM stage (*p* < 0.001) and tumor size >5 cm (*p* < 0.001). However, ALKBH5 overexpression was negatively associated with vascular invasion (*p* = 0.032).

Conclusions: High expression of METTL3 predicted poor outcome. In contrast, high expression of METTL14 was associated with better outcome. Thus, we suggest that among all the m6A regulators, METTL3 and METTL14 could be potential prognostic markers in cancers.

## INTRODUCTION

According to world cancer report 2020, there will be an estimated 60% increase in cancer cases over the next two decades and they will cause about one sixth of deaths worldwide [1]. Although certain progresses have been made in cancer treatment in the past decades, the overall survival of cancer patients is still unsatisfactory. Therefore, biomarkers which can function as prognosticators for the survival time in cancer are necessarily needed. N6-methyladenosine (m6A) modification, an epigenetic modification found in eukaryotes, has been a hot topic in recent years. As the most abundant epigenetic modification in eukaryotes [2], m6A modification is associated with RNA splicing [3–5], maturation [5], stabilization [6] and translation initiation [7]. As a result, m6A modification participates in several biological processes: neural development [8], disease occurrence [9, 10] and tumorigenesis [11–13]. This reversible modification can be added or removed by writers and erasers [14]. Writers are known as m6A methyltransferases, such as METTL3, METTL14, WTAP, KIAA1429 and RBM15/RBM15B. The two major erasers, FTO and ALKBH5, function as m6A demethylases. Furthermore, there are binding proteins called readers [14], represented by YTHDC, IGF2BP and HNRNPC, which recognize specifically modified RNA to exercise different subsequent reactions, including translation and degradation. Recently, emerging studies reported that the above mentioned m6A regulators were of great significance in tumorigenesis [15–17], tumor progression [18, 19] and metastasis [20]. For example, the writer METTL14 could suppress UVB-induced skin tumorigenesis and act as a critical epitranscriptomic mechanism to facilitate global genome repair which is essential for preventing mutagenesis and skin cancer [17]. Moreover, Bo Tang and his colleagues revealed that the eraser ALKBH5 suppressed pancreatic cancer tumorigenesis through promoting transcription of WIF-1 mRNA and inhibiting Wnt signaling pathway in a m6A dependent manner [21]. Additionally, the reader YTHDF1 could promote translation of autophagy-related genes ATG2A and ATG14 by binding to m6A-modified ATG2A and ATG14 mRNA, which facilitated autophagy and autophagy-related human hepatocellular carcinoma progression [15]. YTHDF1 could also enhance ferroptosis by promoting the activation of autophagy and BECN1 mRNA stability in hepatic stellate cells [22]. Overall, there are high-complexity links between m6A and different types of programmed cell death, which are closely related with the initiation, progression and resistance of cancer [23]. Furthermore, there is increasing evidence suggesting that dysregulated expression of m6A regulators exists in major types of cancers and correlates with poor prognosis. However, these survival data were contradictory among different cancer types and regulators, suggesting that a meta-analysis is required to identify prognostic markers. Therefore, in this study, we conducted a systematic review and meta-analysis to assess the prognostic and clinicopathological value of m6A regulators in cancer patients.

## RESULTS

### Study characteristics

**Figure 1:**
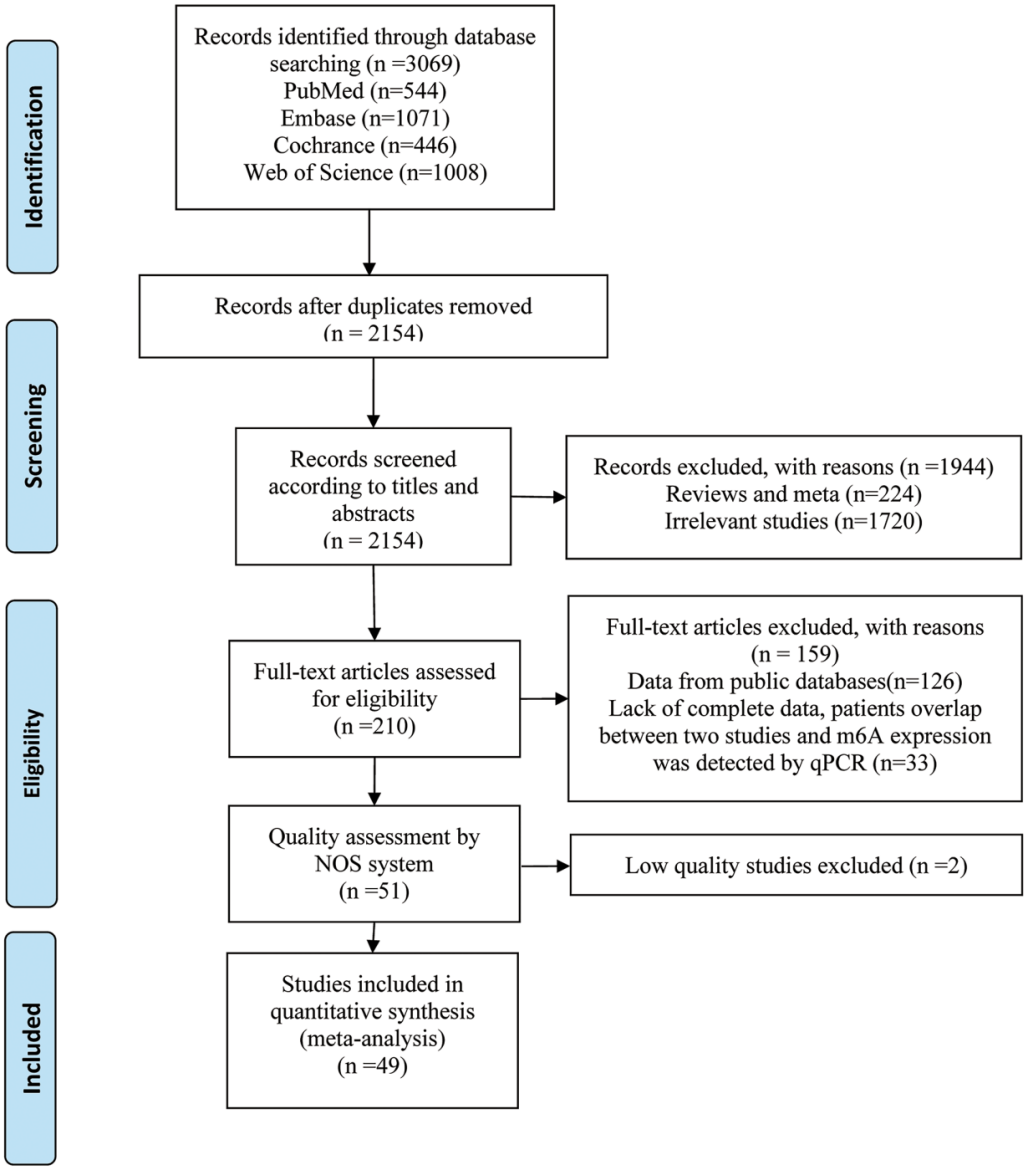
Flow diagram of reviewing and selecting studies.

The literature selection is presented in Figure 1, and the characteristics of eligible studies are shown in Table 1. A total of 3069 relevant studies were retrieved through an initial search. Among them, 915 duplicated records and 1944 unrelated records were excluded based on title or abstract. We subjected 210 studies to full-text screening, of which 159 studies were excluded because they did not meet the inclusion criteria. The remaining 51 articles were further assessed for quality by the Newcastle-Ottawa Scale (NOS) system, and only high-quality studies (NOS ≥ 6) were included in the meta-analysis. Finally, we included 49 cohort studies [6, 15, 24–70] comprising 7006 patients. All studies were published between 2017 and 2022. Forty-eight studies were conducted in Asia and one was conducted in Europe. Sample size ranged from 31 to 603 patients per study. In 49 included studies, 27 studies involved m6A writers, 15 studies referred to erasers and 9 studies were related to readers. The included studies totally reported 20 types of cancers, including digestive system cancer (*n* = 33), respiratory system cancer (*n* = 6), urinary system cancer (*n* = 4), female reproductive system cancer (*n* = 2) and others (*n* = 4). With respect to survival data, 48 studies reported overall survival (OS), 9 studies presented disease-free survival (DFS), and 4 studies showed relapse-free survival (RFS).

**Table 1:** The main characteristics of included studies.

**Study**	**m6A regulators**	**Country**	**Ethnicity**	**Cancer types**	**Follow-up (months)**	**Sample size (M/F)**	**TMN stage**	**Cut-off value**	**Outcome**	**HR and 95% CI**	**NOS score**	**Status**
Yang 2020 (3)	METTL14	China	Asian	Colorectal cancer	NA	37 (27/10)	I–IV	score > 6 (0–12)	RFS	Reported	6	Included
Chen 2020	METTL14	China	Asian	Colorectal cancer	NA	112 (74/38)	I–IV	> median	OS	Reported	7	Included
Wang 2022	METTL14	China	Asian	Colorectal cancer	60	72 (44/28)	I–IV	NA	0S	Reported	7	Included
Deng 2019	METTL3	China	Asian	Colorectal cancer	72–108	181 (97/84)	I–IV	NA	OS	Reported	7	Included
Li 2019 (1)	METTL3	China	Asian	Colorectal carcinoma	80	OS:432 (257/175) DFS:389	NA	> median	OS DFS	OS: Reported DFS: Calculated	6	Included
Shengli 2022	METTL3	China	Asian	Colorectal cancer	60	111 (51/60)	I–IV	score ≥ 4 (0–12)	OS	Calculated	7	Included
Ma 2022	KIAA1429	China	Asian	Colorectal cancer	100	111 (75/36)	I–IV	NA	OS	Calculated	7	Included
Yang 2020 (1)	ALKBH5	China	Asian	Colon cancer	80	60 (25/35)	I–IV	score ≥ 4 (0–12)	OS DFS	Reported	7	Included
Ruan 2021	FTO	China	Asian	Colorectal cancer	140	369 (209/160)	I–III	> median	OS RFS	Reported	6	Included
Nishizawa 2018	YTHDF1	Japan	Asian	Colorectal cancer	NA	63 (41/22)	I–IV	score = 2+ or 3+ (0–3)	OS	Reported	7	Included
Yue 2019	METTL3	China	Asian	Gastric cancer	NA	120 (79/41)	I–IV	NA	OS DFS	Reported	7	Included
Wang 2020	METTL3	China	Asian	Gastric cancer	60	83 (61/22)	I–IV	score > 7 (0-–12)	OS	Reported	6	Included
Yang 2020 (2)	METTL3	China	Asian	Gastric cancer	21-84	OS:196 (131/65) DFS:156	I–IV	score > 145 (0-300)	OS DFS	Reported	8	Included
Sun 2020	METTL3	China	Asian	Gastric cancer	NA	OS:80 RFS:58 (NA)	I–IV	score = 2+ or 3+ (0–3)	OS RFS	Reported	7	Included
Wang 2021 (1)	METTL16	China	Asian	Gastric cancer	49.1	231 (155/76)	I–IV	> median	OS	Reported	8	Included
Liu 2021	METTL14	China	Asian	Gastric cancer	100	248 (183/65)	I–IV	score > 6 (0–12)	OS	Reported	7	Included
Li 2019 (2)	FTO ALKBH5	China	Asian	Gastric cancer	100	450 (308/142)	I–IV	score ≥ 6 (0–12)	OS	Reported	6	Included
Xu 2017	FTO	China	Asian	Gastric cancer	60	128 (68/60)	I–IV	NA	OS	Reported	7	Included
Yuan 2022	YTHDC2	China	Asian	Gastric cancer	80	120 (86/34)	I–IV	NA	OS	Reported	6	Included
Xia 2019	METTL3	China	Asian	Pancreatic cancer	15-26	40 (35/5)	I–III	> median	OS	Calculated	6	Included
Guo 2020	ALKBH5	China	Asian	Pancreatic cancer	60	42 (19/23)	I–III	median	OS	Calculated	7	Included
Zeng 2021	FTO	China	Asian	Pancreatic cancer	NA	50 (27/23)	I–IV	> average	OS	Calculated	8	Included
Tan 2022	FTO	China	Asian	Pancreatic cancer	NA	209 (NA)	I–IV	score > 6 (0–12)	OS	Reported	8	Included
Li 2021	YTHDF1	China	Asian	Hepatocellular carcinoma	60	120 (32/88)	I–III	NA	OS DFS	Reported	7	Included
Ma 2017	METTL14	China	Asian	Hepatocellular carcinoma	NA	220 (193/27)	I–IV	> median	OS RFS	Calculated	3	Not included
Xu 2022 (1)	METTL3	China	Asian	Intrahepatic cholangiocarcinoma	NA	96 (53/43)	I–IV	> median	OS DFS	OS: Reported DFS: Calculated	6	Included
Ye 2020	FTO	China	Asian	Liver cancer	60	309 (NA)	I–III	score ≥ 6 (0–12)	OS	Reported	7	Included
Wang 2021 (2)	METTL3	China	Asian	Oesophageal squamous cell carcinoma	NA	81 (64/17)	I–IV	score > 300 (0–400)	OS	Calculated	7	Included
Xia 2020	METTL3	China	Asian	Esophageal squamous cell carcinoma	108	207 (151/56)	I–IV	score > 8 (0–12)	OS DFS	Reported	7	Included
Nagaki 2020	FTO ALKBH5	Japan	Asian	Esophageal squamous cell carcinoma	41.5–60	177 (153/24)	NA	score = 2+ or 3+ (0–3)	OS	ALKBH5: Reported FTO: Calculated	6	Included
Liu 2020	METTL3	China	Asian	Oral squamous cell carcinoma	3–106	101 (68/33)	I–IV	Youden index	OS	Reported	7	Included
Xu 2021	METTL3	China	Asian	Oral squamous cell carcinoma	80	94 (51/43)	I–IV	score ≥ 4 (0–12)	OS	Calculated	7	Included
Guo 2022	METTL3	China	Asian	Head and neck squamous cell carcinoma	80	100 (99/1)	I–IV	score ≥ 8 (0–12)	OS	Reported	7	Included
Chen 2021	METTL3	China	Asian	Gallbladder-cancer	NA	120 (57/63)	I–IV	> median	OS DFS	Reported	6	Included
Yang 2021	FTO	China	Asian	Lung adenocarcinoma	120	83 (55/28)	I–IV	score ≥ 6 (0–8)	OS	Calculated	8	Included
Huang 2018	ALKBH5	China	Asian	Lung adenocarcinoma	3–125	88 (47/41)	I–IV	HSCORE > 100%	OS	Reported	7	Included
Xu 2022 (2)	YTHDF2	China	Asian	Lung squamous cell carcinoma	60	73 (66/7)	I–III	> median	OS	Reported	7	Included
Tsuchiya 2021	YTHDF1 and YTHDF2	Japan	Asian	Non–small-cell lung cancer	NA	603 (414/189)	I–IV	YTHDF1: score > 118(0-300) YTHDF2: score > 118 (0–300)	OS RFS	Reported	6	Included
Lu 2020	METTL3	China	Asian	Nasopharyngeal carcinoma	10.33–91.67	55 (30/25)	I–IV	score > 3 (0–9)	OS DFS	OS: Reported DFS: Calculated	7	Included
Du 2022	IGF2BP3	China	Asian	Nasopharyngeal carcinoma	150	70 (56/14)	I–IV	NA	OS	Reported	7	Included
Gu 2019	METTL14	China	Asian	Bladder cancer	NA	98 (NA)	NA	NA	OS	Calculated	3	Not included
Han 2019	METTL3	China	Asian	Bladder cancer	60–96	180 (141/39)	I–IV	score > 3 (0–9)	OS	Calculated	7	Included
Yu 2021	ALKBH5	China	Asian	Bladder cancer	60	161 (124/37)	I–IV	score ≥ 8 (0–12)	OS	Calculated	7	Included
Li 2017	METTL3	China	Asian	Renal cell carcinoma	100	145 (89/56)	I–IV	NA	OS	Reported	7	Included
Zhang 2020	ALKBH5	China	Asian	Renal cell carcinoma	100	96 (60/36)	I–IV	score ≥ 8 (0–12)	OS	Calculated	7	Included
Niu 2019	FTO	China	Asian	Breast tumor	96	53 (0/53)	NA	NA	OS	Calculated	7	Included
Hua 2018	METTL3	China	Asian	Ovarian carcinoma	NA	162 (0/162)	I–IV	> median	OS	Reported	8	Included
Lin 2022	METTL3	China	Asian	Thyroid carcinoma	36	80 (25/55)	I–IV	> median	OS	Calculated	6	Included
Orouji E 2020	YTHDF1	Germany	European	Merkel cell carcinoma	NA	31 (NA)	NA	score > 8	OS	Calculated	7	Included
Li 2020	WTAP, KIAA1429, RBM15, RBM15B, METTL3, METTL14, METTL16, HNRNPC, HNRNPA2B1, YTHDF1, YTHDF2, YTHDF3, YTHDC1, FTO, ALKBH5	China	Asian	Osteosarcoma	60	120 (NA)	NA	score > 6	OS	Reported	6	Included
Wei 2022	YTHDF1	China	Asian	Osteosarcoma	60	56 (NA)	I–IV	> median	OS	Calculated	6	Included

Abbreviations: CI: confidence interval; HR: hazard ratio; OS: overall survival; DFS: disease-free survival; RFS: relapse-free survival; NA: not available; F: female; M: male.

### Expression of m6A regulators and prognosis of cancer patients

**Figure 2:**
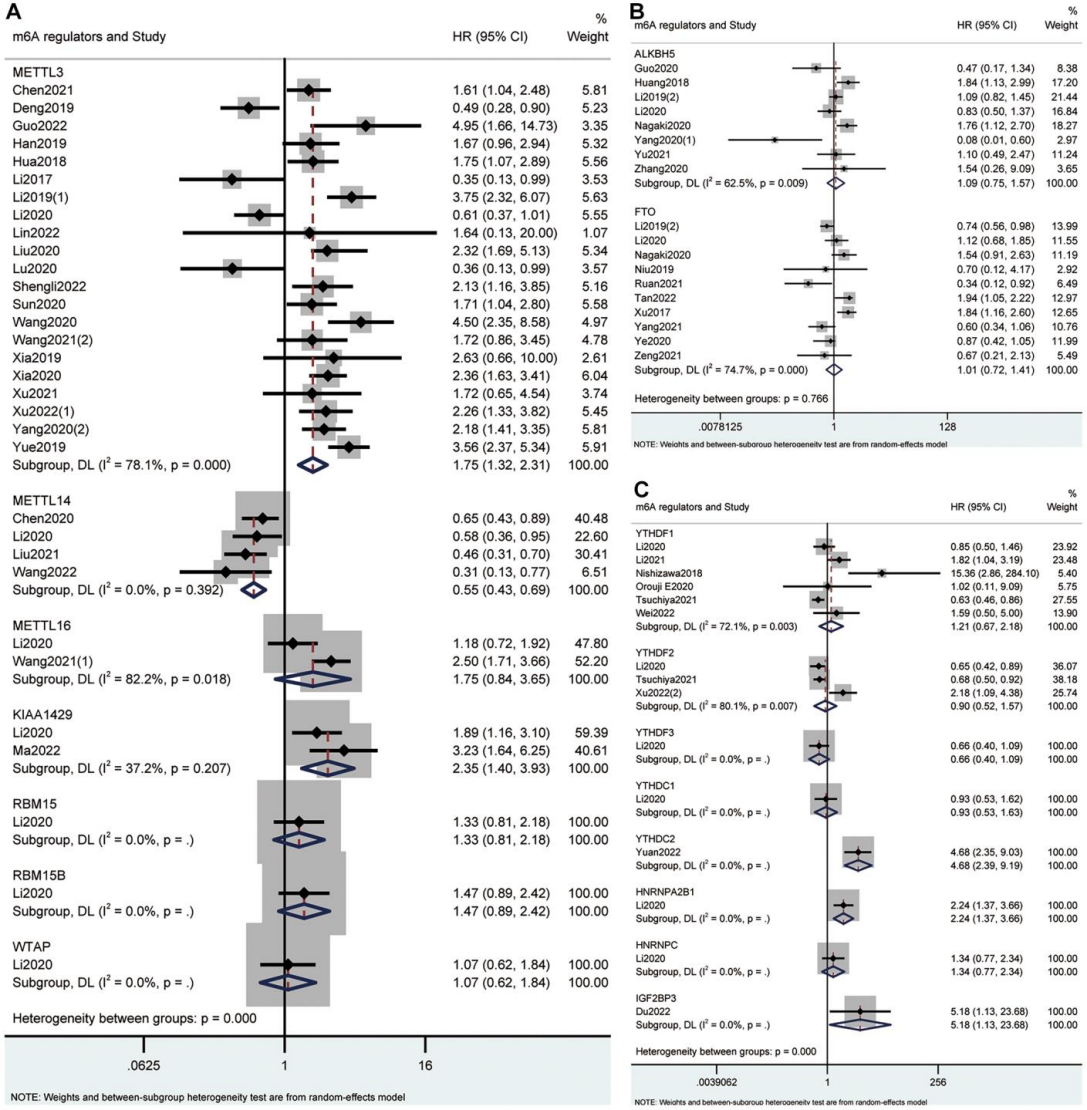
Forest plots for the association of m6A writers (**A**), erasers (**B**) and readers (**C**) with OS in cancer patients.

**Figure 3:**
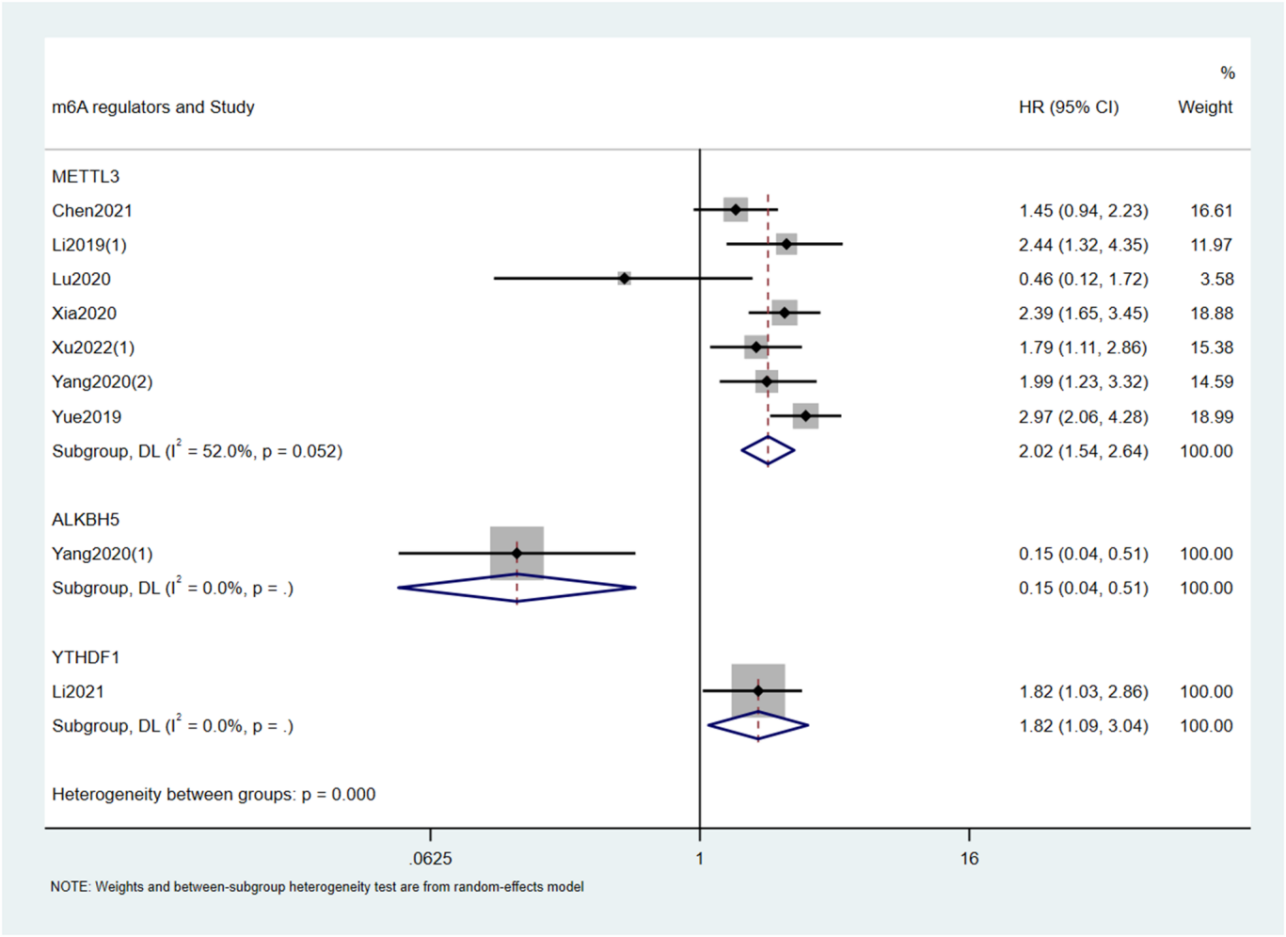
Forest plots for the association of m6A regulators with DFS in cancer patients.

Based on the type of m6A writers, we carried out meta-analysis and found that high expression of METTL3 had an unfavorable effect on OS (HR = 1.75; 95% CI: 1.32–2.31, *p* < 0.001; I^2^ = 78.1%, *p* < 0.001; Figure 2, Table 2) and DFS (HR = 2.02; 95% CI: 1.54–2.64, *p* < 0.001; I^2^ = 52%, *p* = 0.052; Figure 3, Table 2) in cancer patients. Similarly, high expression of KIAA1429 was associated with poor OS (HR = 2.35; 95% CI: 1.40–3.93, *p* = 0.001; I^2^ = 37.2%, *p* = 0.207; Figure 2, Table 2). On the contrary, high expression of METTL14 had a favorable effect on OS (HR = 0.55; 95% CI: 0.43–0.69, *p* < 0.001; I^2^ = 0.0%, *p* = 0.392; Figure 2, Table 2). Furthermore, the expression of METTL16 was not significantly associated with OS in cancer patients (Figure 2, Table 2). Similarly, neither erasers nor readers were significantly associated with OS in cancer patients. (Figure 2, Table 2). We did not perform a meta-analysis of m6A regulators and RFS because there were not enough studies.

**Table 2:** Summary of the meta-analysis of m6A regulators and prognosis in cancer patients.

**Regulators**	**Outcome**	**Studies**	**HR**	**95% Cl**	***P*-value**	**Heterogeneity**		**Effects model**
						**I^2^**	***P*-value**	
METTL3	OS	21	1.75	1.32–2.31	0	78.10%	0	Random
	DFS	7	2.02	1.54–2.64	0	52%	0.052	Random
METTL14	OS	4	0.55	0.43–0.69	0	0.00%	0.392	Random
KIAA1429	OS	2	2.35	1.40–3.93	0.001	37.20%	0.207	Random
METTL16	OS	2	1.75	0.84–3.65	0.137	82.20%	0.018	Random
ALKBH5	OS	8	1.09	0.75–1.57	0.657	62.50%	0.009	Random
FTO	OS	10	1.01	0.72–1.41	0.966	74.70%	0	Random
YTHDF1	OS	6	1.21	0.67–2.18	0.532	72.10%	0.003	Random
YTHDF2	OS	3	0.9	0.52–1.57	0.715	80.10%	0.007	Random

Abbreviations: CI: confidence interval; HR: hazard ratio; OS: overall survival; DFS: disease-free survival.

### Subgroup analysis for different m6A regulators and cancer types

For further exploration, subgroup analyses were conducted according to cancer types. As shown in Table 3, high expression of METTL3 was correlated with poor OS (HR = 2.72; 95% CI: 1.81–4.07, *p* < 0.001; I^2^ = 64.2%, *p* = 0.039) and DFS (HR = 2.58; 95% CI: 1.92–3.47, *p* < 0.001; I^2^ = 37.9%, *p* = 0.205) in gastric cancer. Moreover, high expression of METTL3 was significantly associated with poor OS in esophageal squamous cell carcinoma (HR = 2.20; 95% CI: 1.59–3.05, *p* < 0.001; I^2^ = 0.0%, *p* = 0.436) and oral squamous cell carcinoma (HR = 2.16; 95% CI: 1.33–3.49, *p* = 0.002; I^2^ = 0.0%, *p* = 0.602). However, the expression of METTL3 or METTL14 was not significantly associated with OS in colorectal cancer. The expression of FTO was also not significantly associated with OS in gastric cancer and pancreatic cancer. Furthermore, we did not find a significant association between YTHDF1 and OS in osteosarcoma.

**Table 3:** Subgroup analysis of the correlation between m6A regulators and cancer prognosis based on cancer types.

**Regulators**	**Cancer types**	**Outcome**	**Studies**	**HR**	**95% Cl**	***P*-value**	**Heterogeneity**		**Effects model**
							**I^2^**	***P*-value**	
METTL3	oral squamous cell carcinoma	OS	2	2.16	1.33–3.49	0.002	0.00%	0.602	Fix
	esophageal squamous cell carcinoma	OS	2	2.2	1.59–3.05	0	0.00%	0.436	Fix
	gastric cancer	OS	4	2.72	1.81–4.07	0	64.20%	0.039	Random
	gastric cancer	DFS	2	2.58	1.92–3.47	0	37.90%	0.205	Fix
	colorectal cancer	OS	3	1.59	0.48–5.26	0.448	92.9%,	0	Random
METTL14	colorectal cancer	OS	2	0.51	0.26–1.00	0.051	53.50%	0.142	Random
FTO	pancreatic cancer	OS	2	1.32	0.48–3.60	0.586	65.90%	0.087	Random
	gastric cancer	OS	2	1.15	0.47–2.81	0.756	92.40%	0	Random
YTHDF1	osteosarcoma	OS	2	0.95	0.58–1.54	0.833	0.00%	0.337	Fix

Abbreviations: CI: confidence interval; HR: hazard ratio; OS: overall survival; DFS: disease-free survival.

### Expression of m6A regulators and the clinicopathological parameters

**Figure 4:**
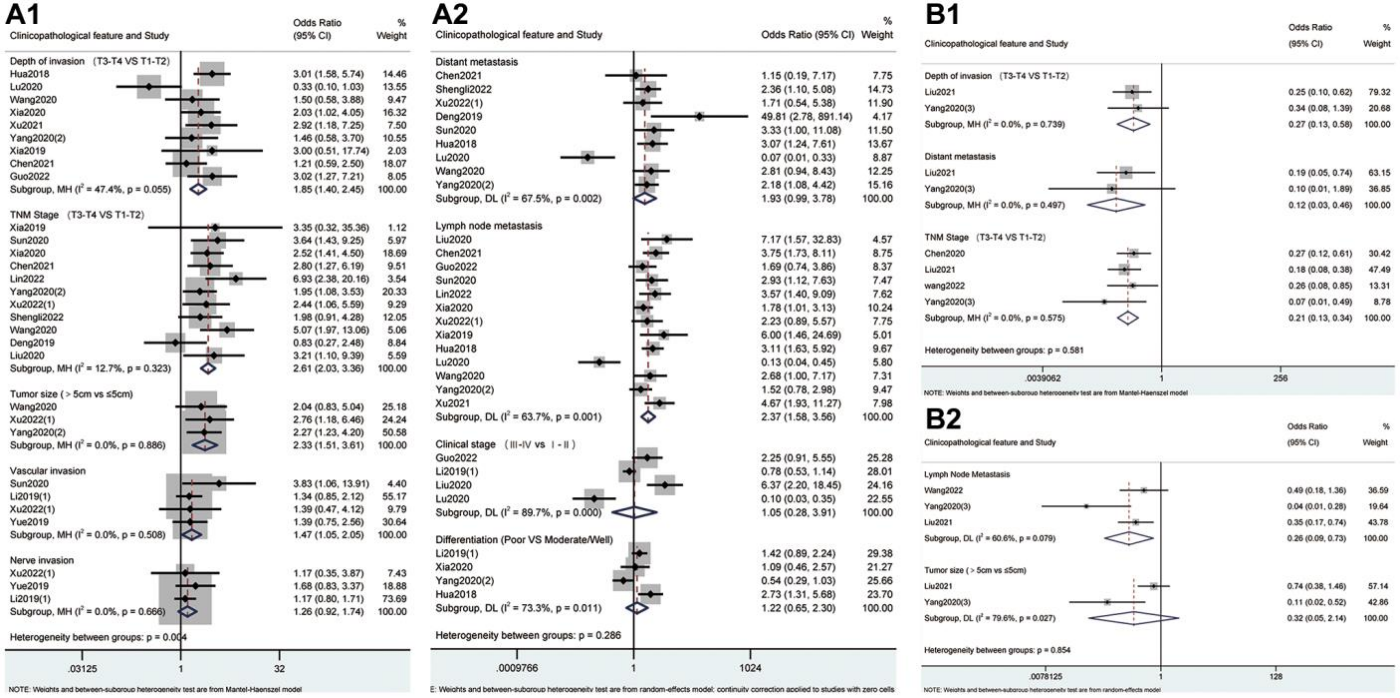
Forest plots for the association of METTL3 (**A**) and METTL14 (**B**) with clinicopathological parameters in cancer patients.

**Figure 5:**
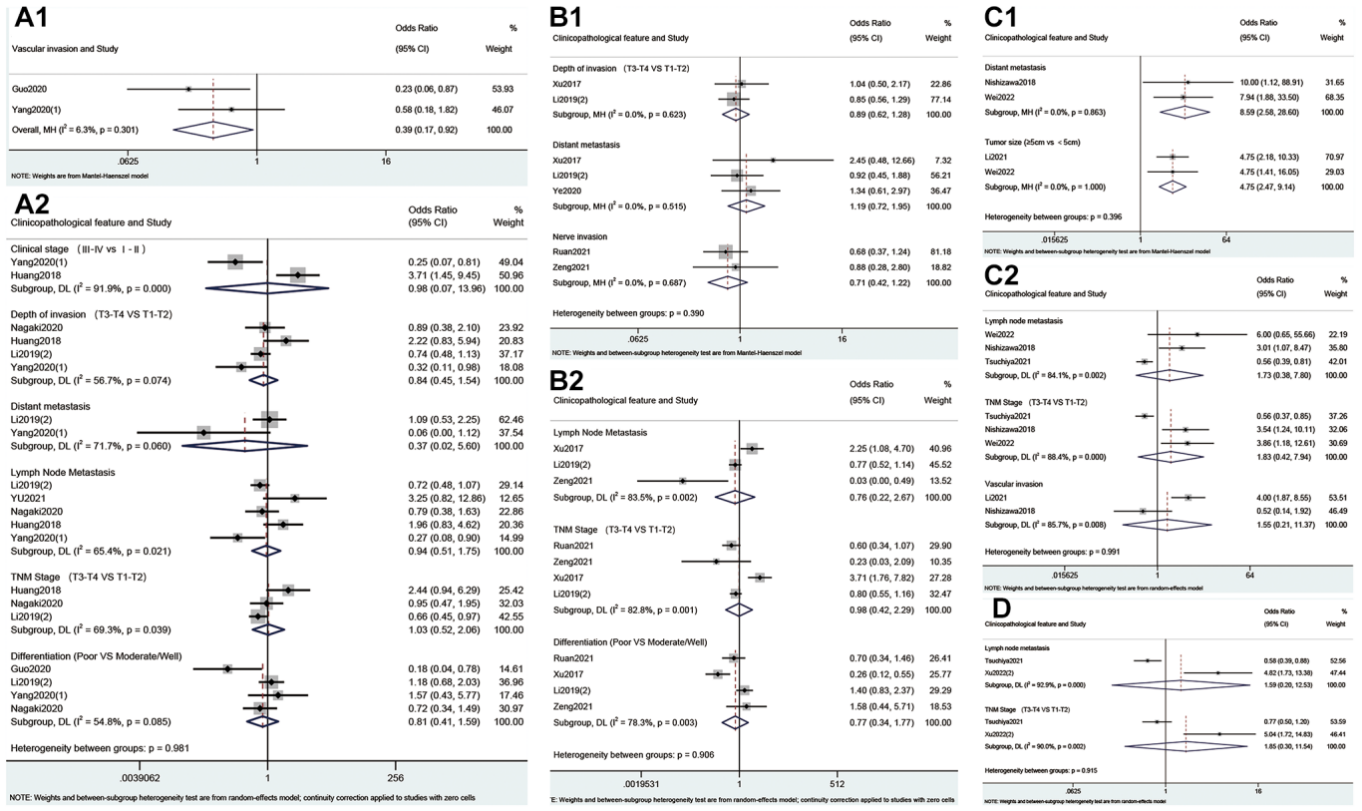
Forest plots for the association of ALKBH5 (**A**), FTO (**B**), YTHDF1 (**C**) and YTHDF2 (**D**) with clinicopathological parameters in cancer patients.

As shown in Figure 4 and Table 4, high expression of METTL3 was associated with advanced pT stage (OR = 1.85; 95% CI: 1.40–2.45, *p* < 0.001; I^2^ = 47.4%, *p* = 0.055), pN stage (OR = 2.37; 95% CI: 1.58–3.56, *p* < 0.001; I^2^ = 63.7%, *p* = 0.001), TNM stage (OR = 2.61; 95% CI: 2.03–3.36, *p* < 0.001; I^2^ = 12.7%, *p* = 0.323), tumor size >5 cm (OR = 2.33; 95% CI: 1.51–3.61, *p* < 0.001; I^2^ = 0.0%, *p* = 0.886) and vascular invasion (OR = 1.47; 95% CI: 1.05–2.05, *p* = 0.024; I^2^ = 0.0%, *p* = 0.508). Conversely, high expression of METTL14 correlated negatively with pT stage (OR = 0.27; 95% CI: 0.13–0.58, *p* = 0.001; I^2^ = 0.0%, *p* = 0.739), pM stage (OR = 0.12; 95% CI: 0.03–0.46, *p* = 0.002; I^2^ = 0.0%, *p* = 0.497), pN stage (OR = 0.26; 95% CI: 0.09–0.73, *p* = 0.011; I^2^ = 60.6%, *p* = 0.079) and TNM stage (OR = 0.21; 95% CI: 0.13–0.34, *p* < 0.001; I^2^ = 0.0%, *p* = 0.575). Meanwhile, there was a statistical association between overexpression of ALKBH5 and negative vascular invasion (OR=0.39; 95%CI: 0.17-0.92, *p* = 0.032; I^2^ = 6.3%, *p* = 0.301, Figure 5). Furthermore, overexpression of YTHDF1 was associated with advanced pM stage (OR = 8.59; 95% CI: 2.58–28.60, *p* < 0.001; I^2^ = 0.0%, *p* = 0.863, Figure 5) and tumor size >5 cm (OR = 4.75; 95% CI: 2.47–9.14, *p* < 0.001; I^2^ = 0.0%, *p* = 1.000, Figure 5).

**Table 4:** The correlations between m6A regulators with clinicopathological characteristics in cancer patients.

**m6A regulator**	**Clinicopathological feature**	**Studies (*n*)**	**Patients (*n*)**	**References**	**OR (95% CI)**	***P* value**	**Heterogeneity**		**Effects model**
							**I² (%)**	***P* value**	
METTL3	Depth of invasion (T3–T4 vs. T1–T2)	9	1057	Hua 2018; Lu 2020; Wang 2020; Xia 2020; Xu 2021; Yang 2020 (2); Xia 2019; Chen 2021; Guo 2022	1.85 (1.40–2.45)	0.000	47.4	0.055	Fix
	Lymph Node Metastasis	13	1421	Liu 2020; Chen 2021; Guo 2022; Sun 2020; Lin 2022; Xia 2020; Xu 2022 (1); Xia 2019; Hua 2018; Lu 2020; Wang 2020; Yang 2020 (2); Xu 2021	2.37 (1.58–3.56)	0.000	63.7	0.001	Random
	TNM Stage (T3–T4 vs. T1–T2)	11	1303	Xia 2019; Sun 2020; Xia 2020; Chen 2021; Lin 2022; Yang 2020 (2); Xu 2022 (1); Shengli 2022; Wang 2020; Deng 2019; Liu 2020	2.61 (2.03–3.36)	0.000	12.7	0.323	Fix
	Tumor size (>5 cm vs ≤ 5 cm)	3	375	Wang 2020; Xu 2022 (1); Yang 2020 (2)	2.33 (1.51–3.61)	0.000	0.0	0.886	Fix
	Vascular invasion	4	781	Sun 2020; Li 2019 (1); Xu 2022 (1); Yue 2019	1.47 (1.05–2.05)	0.024	0.0	0.508	Fix
	Distant metastasis	9	1091	Chen 2021; Shengli 2022; Xu 2022 (1); Deng 2019; Sun 2020; Hua 2018; Lu 2020; Wang 2020; Yang 2020 (2)	1.93 (0.99–3.78)	0.054	67.5	0.002	Random
	Clinical stage III–IV vs. II–II	4	688	Guo 2022; Li 2019 (1); Liu 2020; Lu 2020	1.05 (0.28–3.91)	0.936	89.7	0.000	Random
	Differentiation (Poor vs. Moderate/Well)	4	997	Li 2019 (1); Xia 2020; Yang 2020 (2); Hua 2018	1.22 (0.65–2.30)	0.529	73.3	0.011	Random
	Nerve invasion	3	724	Xu 2022 (1); Yue 2019; Li 2019 (1)	1.26 (0.92–1.74)	0.150	0.0	0.666	Fix
METTL14	Depth of invasion (T3–T4 vs. T1–T2)	2	285	Liu 2021; Yang 2020 (3)	0.27 (0.13–0.58)	0.001	0.0	0.739	Fix
	Lymph Node Metastasis	3	357	Wang 2022; Yang 2020 (3); Liu 2021	0.26 (0.09–0.73)	0.011	60.6	0.079	Random
	Distant metastasis	2	285	Liu 2021; Yang 2020 (3)	0.12 (0.03–0.46)	0.002	0.0	0.497	Fix
	TNM Stage (T3–T4 vs. T1–T2)	4	466	Chen 2020; Liu 2021; Wang 2022; Yang 2020 (3)	0.21 (0.13–0.34)	0.000	0.0	0.575	Fix
	Tumor size (>5 cm vs. ≤5 cm)	2	285	Liu 2021; Yang 2020 (3)	0.32 (0.05–2.14)	0.241	79.6	0.027	Random
ALKBH5	Vascular invasion	2	102	Guo 2020; Yang 2020 (1)	0.39 (0.17–0.92)	0.032	6.3	0.301	Fix
	Clinical stage (III–IV vs. I– II)	2	148	Yang 2020 (1); Huang 2018	0.98 (0.07–13.96)	0.988	91.9	0.000	Random
	Depth of invasion (T3–T4 vs. T1–T2)	4	775	Nagaki 2020; Huang 2018; Li 2019 (2); Yang 2020 (1)	0.84 (0.45–1.54)	0.564	56.7	0.074	Random
	Differentiation (Poor vs. Moderate/Well)	4	729	Guo 2020; Li 2019 (2); Yang 2020 (1); Nagaki 2020	0.81 (0.41–1.59)	0.532	54.8	0.085	Random
	Distant metastasis	2	510	Li 2019 (2); Yang 2020 (1)	0.37 (0.02–5.60)	0.475	71.7	0.060	Random
	Lymph Node Metastasis	5	936	Li 2019 (2); Yu 2021; Nagaki 2020; Huang 2018; Yang 2020 (1)	0.94 (0.51–1.75)	0.851	65.4	0.021	Random
	TNM Stage (T3–T4 vs. T1–T2)	3	715	Huang 2018; Nagaki 2020; Li 2019 (2)	1.03 (0.52–2.06)	0.925	69.3	0.039	Random
FTO	Depth of invasion (T3-T4 vs. T1–T2)	2	578	Xu 2017; Li 2019 (2)	0.89 (0.62–1.28)	0.533	0	0.623	Fix
	Differentiation (Poor vs. Moderate/Well)	4	997	Ruan 2021; Xu 2017; Li 2019 (2); Zeng 2021	0.77 (0.34–1.77)	0.537	78.3	0.003	Random
	Distant metastasis	3	902	Xu 2017; Li 2019 (2); Ye 2020	1.19 (0.72–1.95)	0.502	0	0.515	Fix
	Lymph Node Metastasis	3	628	Xu 2017; Li 2019 (2); Zeng 2021	0.76 (0.22–2.67)	0.671	83.5	0.002	Random
	Nerve invasion	2	419	Ruan 2021; Zeng 2021	0.71 (0.42–1.22)	0.218	0	0.687	Fix
	TNM Stage (T3–T4 vs. T1–T2)	4	997	Ruan 2021; Zeng 2021; Xu 2017; Li 2019 (2)	0.98 (0.42–2.29)	0.969	82.8	0.001	Random
YTHDF1	Distant metastasis	2	113	Nishizawa 2018; Wei 2022	8.59 (2.58–28.60)	0.000	0.0	0.863	Fix
	Tumor size (>5 cm vs ≤5 cm)	2	170	Li 2021; Wei 2022	4.75 (2.47–9.14)	0.000	0.0	1.000	Fix
	Lymph Node Metastasis	3	716	Wei 2022; Nishizawa 2018; Tsuchiya 2021	1.73 (0.38–7.80)	0.476	84.1	0.002	Random
	TNM Stage (T3–T4 vs. T1–T2)	3	716	Wei 2022; Nishizawa 2018; Tsuchiya 2021	1.83 (0.42–7.94)	0.418	88.4	0.000	Random
	Vascular invasion	2	183	Li 2021; Nishizawa 2018	1.55 (0.21–11.37)	0.665	85.7	0.008	Random
YTHDF2	Lymph Node Metastasis	2	676	Tsuchiya 2021; Xu 2022 (2)	1.59 (0.20–12.53)	0.660	92.9	0.000	Random
	TNM Stage (T3–T4 vs. T1–T2)	2	676	Tsuchiya 2021; Xu 2022 (2)	1.85 (0.30–11.54)	0.512	90.0	0.002	Random

Abbreviations: CI: confidence interval; OR: odds ratio.

### Sensitivity analysis

**Figure 6:**
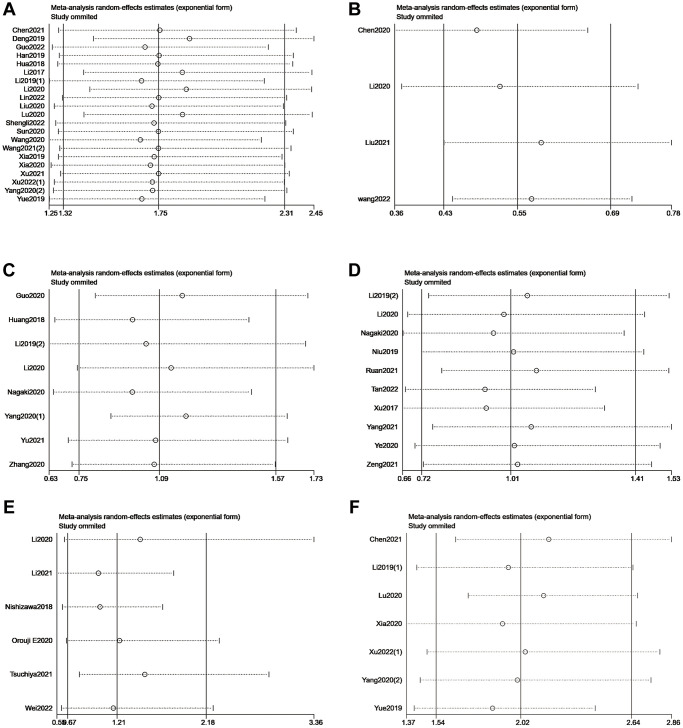
Sensitivity analysis of METTL3 (**A**), METTL14 (**B**), ALKBH5 (**C**), FTO (**D**), and YTHDF1 (**E**) for OS. Sensitivity analysis of METTL3 (**F**) for DFS.

We omitted individual studies successively to estimate the impact of each study in our meta-analysis. No individual study modified the pooled HR of included studies reporting OS or DFS significantly, which proved that the results were stable (Figure 6).

### Publication bias

**Figure 7:**
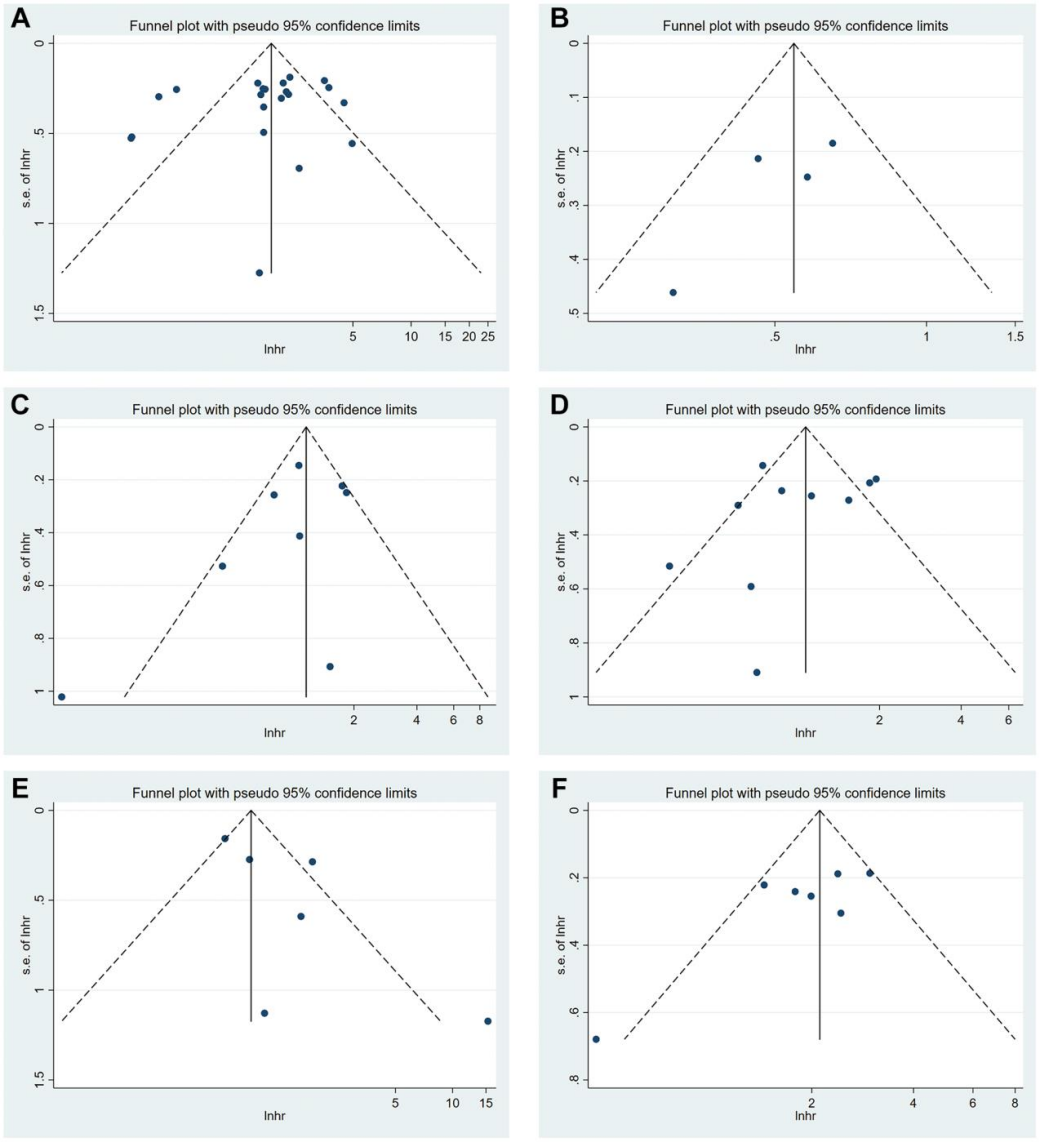
Funnel plot of METTL3 (**A**), METTL14 (**B**), ALKBH5 (**C**), FTO (**D**) and YTHDF1 (**E**) for OS. Funnel plot of METTL3 (**F**) for DFS.

Funnel plots were generated to detect publication bias (Figure 7). The studies were distributed uniformly around the axis, indicating no obvious publication bias. Meanwhile, no obvious publication bias was found according to Begg’s test and Egger’s test (Table 5).

**Table 5:** Publication bias test of included studies in our meta-analysis.

**Regulators**	**Outcome**	**Begg’s test (*P* value)**	**Egger’s test (*P* value)**
METTL3	OS	0.415	0.319
METTL4	OS	0.308	0.229
ALKBH5	OS	0.174	0.290
FTO	OS	0.592	0.571
YTHDF1	OS	0.260	0.117
METTL3	DFS	0.230	0.083

Abbreviations: OS: overall survival; DFS: disease-free survival.

## DISCUSSION

m6A modification, a reversible epigenetic modification regulated by three types of proteins (writers, erasers and readers), plays a complicated role in cancer initiation and development [14, 71, 72]. Recent studies have explored how m6A regulators influenced the prognosis of cancer patients. However, results were frequently inconsistent among different cancer types. Therefore, a comprehensive study to summarize the results from current publications is necessary. To report prognostic value of m6A regulators in cancer patients, we analyzed the survival time and clinicopathological features of 7006 patients from 49 studies who expressed different levels of m6A regulators. Results showed that expression level of m6A writers was related to cancer prognosis. In addition, different m6A writers had opposite associations with the prognosis and clinicopathological features in cancer patients. According to the results, there was a possible trend for poor OS and DFS in patients with the high expression of METTL3. Similarly, previous bioinformatic analysis from databases like TCGA, GEO and HPA, supported that high expression of METTL3 was correlated with unfavorable prognosis in various cancers, including gastric cancer [73], colorectal cancer [74], liver cancer [75], prostate cancer [76] and glioma [77]. In most of these databases, RNA-seq was used to detect the level of METTL3. Moreover, a previous meta-analysis including 9 studies showed that high METTL3 expression was associated with poor prognosis in cancer patients, and the expression of METTL3 in included 9 studies were all detected by qRT-PCR. While in the studies included in our analysis, METTL3 was detected only by IHC staining. Combining our studies with the results from databases, we can conclude that METTL3 is related to cancer prognosis at protein level, which strongly suggests that it could be a prognostic predictor. Additionally, this tendency was more prominent in gastric cancer. Previous studies indicated that in human gastric cancer cells, high expression of METTL3 stimulates the expression of GLUT4 and ENO2 via the METTL3/HDGF axis, thereby promoting tumor angiogenesis and glycolysis [6]. Moreover, Ben Yue et al. unveiled that METTL3 stabilized ZMYM1 mRNA in gastric cancer cells, which facilitated EMT and metastasis by repressing E-cadherin promoter [26]. These might account, at least to some extent, for the poor survival of patients with gastric cancer. Furthermore, aberrant expression of METTL3 was involved in the dysfunction of cellular signaling pathways, such as MAPK [74], JAK/STAT [78], PI3K/AKT [79, 80] and Wnt/β-catenin [81] cascades, which are involved in tumor progression, metastasis, migration and stemness. We also found that high expression of METTL3 was associated with advanced TNM stage and pT stage, pN stage, tumor size >5 cm and vascular invasion respectively. Therefore, based on these current results, we believe that METTL3 plays an important role in multiple stages of cancer progression and ultimately affects prognosis. Interestingly, in contrast to METTL3, METTL14, another m6A methylation writer, might be a positive prognosticator. Previous studies have shown that METTL14 might have various functions that have not been fully identified yet, thus its role in cancer remained controversial [82]. In this study, our result confirmed that high level of METTL14 was associated with better OS. Different studies have reported that METTL14 suppressed progression and metastasis in several cancers, such as colorectal cancer [83] and hepatocellular carcinoma [84]. Besides, Panneerdoss et al. found that in METTL14-silenced breast cancer cells, RhoA and PI3K-AKT signaling pathways were highly enriched, which are well-known to be mediators of cancer progression and angiogenesis [85]. Moreover, our study showed that high expression of METTL14 was inversely associated with poor TNM stage, pT stage, pN stage and pM stage. Combining the results of other studies and ours, we inferred that METTL14 plays a role in cancer suppression and could be a favorable index of cancer progression and prognosis. Moreover, METTL3 and METTL14 show completely contrary effects on cancer progression, indicating that METTL3 and METTL14 may have some biological functions that are independent of m6A modification, which deserves further study.

Besides, from the analysis results of clinicopathological features, high expression of YTHDF1 was associated with advanced pM stage and tumor size >5 cm, while high expression of ALKBH5 was negatively associated with vascular invasion. Consistently, a recent study reported that YTHDF1 regulates CRC tumorigenesis and metastasis by promoting ARHGEF2 translation and RhoA signaling in colorectal cancer [20]. High YTHDF1 level is significantly associated with metastatic gene signature in colorectal cancer, while YTHDF1-knockout mice inhibited tumor growth *in vivo* [20]. Therefore, targeting the YTHDF1-m 6 A-ARHGEF2 axis may be a promising therapeutic strategy to inhibit tumor growth, invasion, and metastasis. In addition, ALKBH5, as the second m6A demethylated enzyme discovered after FTO, was reported to promote tumor stem formation in gliomas and promote tumor progression in breast cancer, colon cancer and hepatocellular carcinoma [85, 86]. Conversely, ALKBH5 could inhibit tumor growth in bladder cancer and pancreatic cancer. These findings suggest the complexity of the action of ALKBH5 in cancers. However, no significant relationship was found between high expressions of m6A erasers or readers and poor prognosis. Limitation of sample size and a certain degree of heterogeneity may partly account for this. Additionally, the mechanisms of m6A modification and cancers are complicated [87]. Therefore, more studies are needed to provide further mechanistic insights.

To the best of our knowledge, this is the first study to conduct a meta-analysis of the association between m6A regulators and the prognosis and clinicopathology in cancer patients systematically. Nonetheless, there are still several limitations in our meta-analysis. First, several original data were not available, therefore we had to extract data from the Kaplan-Meier survival curves and this might increase the inaccuracy in our study. Secondly, the ethnicity of included patients was mostly Asian, which may increase the population selection bias. Thirdly, IHC was adopted to detect the expression of m6A regulators in all studies, but the IHC protocols, antibodies and cut-off values were not consistent across the included studies, which may have led to significant heterogeneity between included studies. Therefore, future research should standardize the cut-off values for the expression of m6A regulators, detection antibodies used and IHC staining protocols to better compare the results of different studies. In summary, our meta-analysis provides evidence that the expression level of m6A writers is related to cancer progression and prognosis. Different m6A writer proteins play different roles in patients’ outcome: high expression level of METTL3 is significantly associated with poor prognosis, while high expression of METTL14 leads to better survival rate. Both m6A regulators possess a great potential to become practicable prognosticators in various cancers. Meanwhile, future studies with more complete and representative datasets are required for further exploration.

## METHODS

### Literature search

Relevant articles published up to April 2022 were obtained from PubMed, Embase, Web of Science and the Cochrane library. There were no restrictions on language or date of publication. “N (6)-methyladenosine” and “cancers” were the two main key words we used. The comprehensive search strategy for each database is provided in Supplementary Table 1. All references were managed using EndNote X9. Three reviewers independently analyzed search results. Any disagreements between reviewers were resolved by discussion.

### Inclusion and exclusion criteria

The process of selecting eligible studies was conducted by three reviewers independently. Articles were included when they met the following inclusion criteria: (1) the text evaluated the relation between m6A regulators expression and cancer prognosis; (2) HR and 95% CI were reported or could be calculated from the text; (3) original research; (4) the expression of m6A regulators in tissues was detected by immunohistochemistry; (5) patients were confirmed cancers definitively. The exclusion criteria were: (1) reviews, letters, meeting abstracts; (2) nonhuman studies; (3) sample cases were from databases; (4) duplicate data; (5) studies did not provide necessary and complete data.

### Data extraction and quality assessment

The following information were extracted from eligible studies independently by three researchers: author, published year, country, m6A regulators, cancer types, cancer stage, sample size, gender, cut-off value of m6A regulators and survival data including OS, DFS and RFS. The HR with its 95% CI were extracted from the text directly or calculated from Kaplan-Meier survival curve using Engauge Digitizer. The quality of the included studies was evaluated using the Newcastle Ottawa Scale (NOS) criteria. NOS scores range from 0 to 9. It would be considered as high-quality study if score was more than 5; otherwise, it would be considered as low-quality study. Only studies with NOS ≥ 6 were finally selected for inclusion in meta-analysis. Disagreements were resolved by discussion.

### Statistical analysis

The pooled HR and 95% CI were used to evaluate the relation between m6A regulators and cancer prognosis (OS, DFS and RFS). The pooled odds ratio (OR) and 95%CI were used to evaluate the relationship between m6A regulators and clinicopathological parameters. HRs or ORs >1 represented a poor prognosis in cancer. Heterogeneity among the studies was evaluated by Coltrane’s Q statistic and the I^2^ statistic. If a *p* < 0.1 or I^2^ > 50%, we applied a random-effect model. Otherwise, a fixed-effect model was applied. Subgroup analysis was conducted according to cancer types. In the sensitivity analysis, we omitted individual studies successively to estimate the impact of each study in our meta-analysis. Begg’s test and Egger’s test were used to evaluate publication bias. A two-tailed *p* value < 0.05 was considered statistically significant in all statistical tests. All data analyses were performed using StataSE15.1 (Stata Corporation, College Station, TX, USA).

## Supplementary Materials

Supplementary Table 1

## References

[1] World Cancer Report: Cancer Research for Cancer Prevention. https://publications.iarc.fr/586.

[2] Jia G, Fu Y, He C. Reversible RNA adenosine methylation in biological regulation. Trends Genet. 2013; 29:108–15. 10.1016/j.tig.2012.11.003 23218460 PMC3558665

[3] Liu N, Zhou KI, Parisien M, Dai Q, Diatchenko L, Pan T. N6-methyladenosine alters RNA structure to regulate binding of a low-complexity protein. Nucleic Acids Res. 2017; 45:6051–63. 10.1093/nar/gkx141 28334903 PMC5449601

[4] Ping XL, Sun BF, Wang L, Xiao W, Yang X, Wang WJ, Adhikari S, Shi Y, Lv Y, Chen YS, Zhao X, Li A, Yang Y, et al. Mammalian WTAP is a regulatory subunit of the RNA N6-methyladenosine methyltransferase. Cell Res. 2014; 24:177–89. 10.1038/cr.2014.3 24407421 PMC3915904

[5] Liu N, Dai Q, Zheng G, He C, Parisien M, Pan T. N(6)-methyladenosine-dependent RNA structural switches regulate RNA-protein interactions. Nature. 2015; 518:560–4. 10.1038/nature14234 25719671 PMC4355918

[6] Wang Q, Chen C, Ding Q, Zhao Y, Wang Z, Chen J, Jiang Z, Zhang Y, Xu G, Zhang J, Zhou J, Sun B, Zou X, Wang S. METTL3-mediated m^6^A modification of HDGF mRNA promotes gastric cancer progression and has prognostic significance. Gut. 2020; 69:1193–205. 10.1136/gutjnl-2019-319639 31582403

[7] Tanabe A, Tanikawa K, Tsunetomi M, Takai K, Ikeda H, Konno J, Torigoe T, Maeda H, Kutomi G, Okita K, Mori M, Sahara H. RNA helicase YTHDC2 promotes cancer metastasis via the enhancement of the efficiency by which HIF-1α mRNA is translated. Cancer Lett. 2016; 376:34–42. 10.1016/j.canlet.2016.02.022 26996300

[8] Livneh I, Moshitch-Moshkovitz S, Amariglio N, Rechavi G, Dominissini D. The m^6^A epitranscriptome: transcriptome plasticity in brain development and function. Nat Rev Neurosci. 2020; 21:36–51. 10.1038/s41583-019-0244-z 31804615

[9] Maity A, Das B. N6-methyladenosine modification in mRNA: machinery, function and implications for health and diseases. FEBS J. 2016; 283:1607–30. 10.1111/febs.13614 26645578

[10] Yang Y, Shen F, Huang W, Qin S, Huang JT, Sergi C, Yuan BF, Liu SM. Glucose Is Involved in the Dynamic Regulation of m6A in Patients With Type 2 Diabetes. J Clin Endocrinol Metab. 2019; 104:665–73. 10.1210/jc.2018-00619 30137347

[11] Chen S, Li Y, Zhi S, Ding Z, Wang W, Peng Y, Huang Y, Zheng R, Yu H, Wang J, Hu M, Miao J, Li J. WTAP promotes osteosarcoma tumorigenesis by repressing HMBOX1 expression in an m^6^A-dependent manner. Cell Death Dis. 2020; 11:659. 10.1038/s41419-020-02847-6 32814762 PMC7438489

[12] Li Z, Weng H, Su R, Weng X, Zuo Z, Li C, Huang H, Nachtergaele S, Dong L, Hu C, Qin X, Tang L, Wang Y, et al. FTO Plays an Oncogenic Role in Acute Myeloid Leukemia as a N^6^-Methyladenosine RNA Demethylase. Cancer Cell. 2017; 31:127–41. 10.1016/j.ccell.2016.11.017 28017614 PMC5234852

[13] Shen C, Sheng Y, Zhu AC, Robinson S, Jiang X, Dong L, Chen H, Su R, Yin Z, Li W, Deng X, Chen Y, Hu YC, et al. RNA Demethylase ALKBH5 Selectively Promotes Tumorigenesis and Cancer Stem Cell Self-Renewal in Acute Myeloid Leukemia. Cell Stem Cell. 2020; 27:64–80.e9. 10.1016/j.stem.2020.04.009 32402250 PMC7335338

[14] Cao G, Li HB, Yin Z, Flavell RA. Recent advances in dynamic m6A RNA modification. Open Biol. 2016; 6:160003. 10.1098/rsob.160003 27249342 PMC4852458

[15] Li Q, Ni Y, Zhang L, Jiang R, Xu J, Yang H, Hu Y, Qiu J, Pu L, Tang J, Wang X. HIF-1α-induced expression of m6A reader YTHDF1 drives hypoxia-induced autophagy and malignancy of hepatocellular carcinoma by promoting ATG2A and ATG14 translation. Signal Transduct Target Ther. 2021; 6:76. 10.1038/s41392-020-00453-8 33619246 PMC7900110

[16] Cui YH, Yang S, Wei J, Shea CR, Zhong W, Wang F, Shah P, Kibriya MG, Cui X, Ahsan H, He C, He YY. Autophagy of the m^6^A mRNA demethylase FTO is impaired by low-level arsenic exposure to promote tumorigenesis. Nat Commun. 2021; 12:2183. 10.1038/s41467-021-22469-6 33846348 PMC8041927

[17] Yang Z, Yang S, Cui YH, Wei J, Shah P, Park G, Cui X, He C, He YY. METTL14 facilitates global genome repair and suppresses skin tumorigenesis. Proc Natl Acad Sci U S A. 2021; 118:e2025948118. 10.1073/pnas.2025948118 34452996 PMC8536359

[18] Tao L, Mu X, Chen H, Jin D, Zhang R, Zhao Y, Fan J, Cao M, Zhou Z. FTO modifies the m6A level of MALAT and promotes bladder cancer progression. Clin Transl Med. 2021; 11:e310. 10.1002/ctm2.310 33634966 PMC7851431

[19] Ma L, Xue X, Zhang X, Yu K, Xu X, Tian X, Miao Y, Meng F, Liu X, Guo S, Qiu S, Wang Y, Cui J, et al. The essential roles of m^6^A RNA modification to stimulate ENO1-dependent glycolysis and tumorigenesis in lung adenocarcinoma. J Exp Clin Cancer Res. 2022; 41:36. 10.1186/s13046-021-02200-5 35078505 PMC8788079

[20] Wang S, Gao S, Zeng Y, Zhu L, Mo Y, Wong CC, Bao Y, Su P, Zhai J, Wang L, Soares F, Xu X, Chen H, et al. N6-Methyladenosine Reader YTHDF1 Promotes ARHGEF2 Translation and RhoA Signaling in Colorectal Cancer. Gastroenterology. 2022; 162:1183–96. 10.1053/j.gastro.2021.12.269 34968454

[21] Tang B, Yang Y, Kang M, Wang Y, Wang Y, Bi Y, He S, Shimamoto F. m^6^A demethylase ALKBH5 inhibits pancreatic cancer tumorigenesis by decreasing WIF-1 RNA methylation and mediating Wnt signaling. Mol Cancer. 2020; 19:3. 10.1186/s12943-019-1128-6 31906946 PMC6943907

[22] Shen M, Li Y, Wang Y, Shao J, Zhang F, Yin G, Chen A, Zhang Z, Zheng S. N^6^-methyladenosine modification regulates ferroptosis through autophagy signaling pathway in hepatic stellate cells. Redox Biol. 2021; 47:102151. 10.1016/j.redox.2021.102151 34607160 PMC8495178

[23] Liu L, Li H, Hu D, Wang Y, Shao W, Zhong J, Yang S, Liu J, Zhang J. Insights into N6-methyladenosine and programmed cell death in cancer. Mol Cancer. 2022; 21:32. 10.1186/s12943-022-01508-w 35090469 PMC8796496

[24] Zhang X, Wang F, Wang Z, Yang X, Yu H, Si S, Lu J, Zhou Z, Lu Q, Wang Z, Yang H. ALKBH5 promotes the proliferation of renal cell carcinoma by regulating AURKB expression in an m^6^A-dependent manner. Ann Transl Med. 2020; 8:646. 10.21037/atm-20-3079 32566583 PMC7290639

[25] Zeng J, Zhang H, Tan Y, Wang Z, Li Y, Yang X. m6A demethylase FTO suppresses pancreatic cancer tumorigenesis by demethylating *PJA2* and inhibiting Wnt signaling. Mol Ther Nucleic Acids. 2021; 25:277–92. 10.1016/j.omtn.2021.06.005 34484859 PMC8385122

[26] Yue B, Song C, Yang L, Cui R, Cheng X, Zhang Z, Zhao G. METTL3-mediated N6-methyladenosine modification is critical for epithelial-mesenchymal transition and metastasis of gastric cancer. Mol Cancer. 2019; 18:142. 10.1186/s12943-019-1065-4 31607270 PMC6790244

[27] Yuan W, Chen S, Li B, Han X, Meng B, Zou Y, Chang S. The N6-methyladenosine reader protein YTHDC2 promotes gastric cancer progression via enhancing YAP mRNA translation. Transl Oncol. 2022; 16:101308. 10.1016/j.tranon.2021.101308 34911015 PMC8681016

[28] Yu H, Yang X, Tang J, Si S, Zhou Z, Lu J, Han J, Yuan B, Wu Q, Lu Q, Yang H. ALKBH5 Inhibited Cell Proliferation and Sensitized Bladder Cancer Cells to Cisplatin by m6A-CK2α-Mediated Glycolysis. Mol Ther Nucleic Acids. 2020; 23:27–41. 10.1016/j.omtn.2020.10.031 33376625 PMC7744648

[29] Ye Z, Wang S, Chen W, Zhang X, Chen J, Jiang J, Wang M, Zhang L, Xuan Z. Fat mass and obesity-associated protein promotes the tumorigenesis and development of liver cancer. Oncol Lett. 2020; 20:1409–17. 10.3892/ol.2020.11673 32724383 PMC7377176

[30] Yang X, Zhang S, He C, Xue P, Zhang L, He Z, Zang L, Feng B, Sun J, Zheng M. METTL14 suppresses proliferation and metastasis of colorectal cancer by down-regulating oncogenic long non-coding RNA XIST. Mol Cancer. 2020; 19:46. 10.1186/s12943-020-1146-4 32111213 PMC7047419

[31] Yang X, Shao F, Guo D, Wang W, Wang J, Zhu R, Gao Y, He J, Lu Z. WNT/β-catenin-suppressed FTO expression increases m^6^A of c-Myc mRNA to promote tumor cell glycolysis and tumorigenesis. Cell Death Dis. 2021; 12:462. 10.1038/s41419-021-03739-z 33966037 PMC8106678

[32] Yang P, Wang Q, Liu A, Zhu J, Feng J. ALKBH5 Holds Prognostic Values and Inhibits the Metastasis of Colon Cancer. Pathol Oncol Res. 2020; 26:1615–23. 10.1007/s12253-019-00737-7 31506804

[33] Yang DD, Chen ZH, Yu K, Lu JH, Wu QN, Wang Y, Ju HQ, Xu RH, Liu ZX, Zeng ZL. METTL3 Promotes the Progression of Gastric Cancer via Targeting the MYC Pathway. Front Oncol. 2020; 10:115. 10.3389/fonc.2020.00115 32175271 PMC7054453

[34] Xu QC, Tien YC, Shi YH, Chen S, Zhu YQ, Huang XT, Huang CS, Zhao W, Yin XY. METTL3 promotes intrahepatic cholangiocarcinoma progression by regulating IFIT2 expression in an m^6^A-YTHDF2-dependent manner. Oncogene. 2022; 41:1622–33. 10.1038/s41388-022-02185-1 35094011 PMC8913368

[35] Xu P, Hu K, Zhang P, Sun ZG, Zhang N. Hypoxia-mediated YTHDF2 overexpression promotes lung squamous cell carcinoma progression by activation of the mTOR/AKT axis. Cancer Cell Int. 2022; 22:13. 10.1186/s12935-021-02368-y 34996459 PMC8742419

[36] Xu L, Li Q, Wang Y, Wang L, Guo Y, Yang R, Zhao N, Ge N, Wang Y, Guo C. m^6^A methyltransferase METTL3 promotes oral squamous cell carcinoma progression through enhancement of IGF2BP2-mediated SLC7A11 mRNA stability. Am J Cancer Res. 2021; 11:5282–98. 34873461 PMC8640804

[37] Xu D, Shao W, Jiang Y, Wang X, Liu Y, Liu X. FTO expression is associated with the occurrence of gastric cancer and prognosis. Oncol Rep. 2017; 38:2285–92. 10.3892/or.2017.5904 28849183

[38] Xia TL, Yan SM, Yuan L, Zeng MS. Upregulation of METTL3 Expression Predicts Poor Prognosis in Patients with Esophageal Squamous Cell Carcinoma. Cancer Manag Res. 2020; 12:5729–37. 10.2147/CMAR.S245019 32765076 PMC7367742

[39] Xia T, Wu X, Cao M, Zhang P, Shi G, Zhang J, Lu Z, Wu P, Cai B, Miao Y, Jiang K. The RNA m6A methyltransferase METTL3 promotes pancreatic cancer cell proliferation and invasion. Pathol Res Pract. 2019; 215:152666. 10.1016/j.prp.2019.152666 31606241

[40] Wei K, Gao Y, Wang B, Qu YX. Methylation recognition protein YTH N6-methyladenosine RNA binding protein 1 (YTHDF1) regulates the proliferation, migration and invasion of osteosarcoma by regulating m6A level of CCR4-NOT transcription complex subunit 7 (CNOT7). Bioengineered. 2022; 13:5236–50. 10.1080/21655979.2022.2037381 35156522 PMC8973933

[41] Wang XK, Zhang YW, Wang CM, Li B, Zhang TZ, Zhou WJ, Cheng LJ, Huo MY, Zhang CH, He YL. METTL16 promotes cell proliferation by up-regulating cyclin D1 expression in gastric cancer. J Cell Mol Med. 2021; 25:6602–17. 10.1111/jcmm.16664 34075693 PMC8278090

[42] Wang W, Shao F, Yang X, Wang J, Zhu R, Yang Y, Zhao G, Guo D, Sun Y, Wang J, Xue Q, Gao S, Gao Y, et al. METTL3 promotes tumour development by decreasing APC expression mediated by APC mRNA N^6^-methyladenosine-dependent YTHDF binding. Nat Commun. 2021; 12:3803. 10.1038/s41467-021-23501-5 34155197 PMC8217513

[43] Wang H, Wei W, Zhang ZY, Liu Y, Shi B, Zhong W, Zhang HS, Fang X, Sun CL, Wang JB, Liu LX. TCF4 and HuR mediated-METTL14 suppresses dissemination of colorectal cancer via N6-methyladenosine-dependent silencing of ARRDC4. Cell Death Dis. 2021; 13:3. 10.1038/s41419-021-04459-0 34916487 PMC8677753

[44] Tsuchiya K, Yoshimura K, Inoue Y, Iwashita Y, Yamada H, Kawase A, Watanabe T, Tanahashi M, Ogawa H, Funai K, Shinmura K, Suda T, Sugimura H. YTHDF1 and YTHDF2 are associated with better patient survival and an inflamed tumor-immune microenvironment in non-small-cell lung cancer. Oncoimmunology. 2021; 10:1962656. 10.1080/2162402X.2021.1962656 34408926 PMC8366544

[45] Tan Z, Shi S, Xu J, Liu X, Lei Y, Zhang B, Hua J, Meng Q, Wang W, Yu X, Liang C. RNA N6-methyladenosine demethylase FTO promotes pancreatic cancer progression by inducing the autocrine activity of PDGFC in an m^6^A-YTHDF2-dependent manner. Oncogene. 2022; 41:2860–72. 10.1038/s41388-022-02306-w 35422475 PMC9106577

[46] Sun Y, Li S, Yu W, Zhao Z, Gao J, Chen C, Wei M, Liu T, Li L, Liu L. N^6^-methyladenosine-dependent pri-miR-17-92 maturation suppresses PTEN/TMEM127 and promotes sensitivity to everolimus in gastric cancer. Cell Death Dis. 2020; 11:836. 10.1038/s41419-020-03049-w 33037176 PMC7547657

[47] Pan S, Deng Y, Fu J, Zhang Y, Zhang Z, Qin X. N6-methyladenosine upregulates miR-181d-5p in exosomes derived from cancer-associated fibroblasts to inhibit 5-FU sensitivity by targeting NCALD in colorectal cancer. Int J Oncol. 2022; 60:14. 10.3892/ijo.2022.5304 35014676 PMC8759347

[48] Ruan DY, Li T, Wang YN, Meng Q, Li Y, Yu K, Wang M, Lin JF, Luo LZ, Wang DS, Lin JZ, Bai L, Liu ZX, et al. FTO downregulation mediated by hypoxia facilitates colorectal cancer metastasis. Oncogene. 2021; 40:5168–81. 10.1038/s41388-021-01916-0 34218271 PMC8376648

[49] Orouji E, Peitsch WK, Orouji A, Houben R, Utikal J. Oncogenic Role of an Epigenetic Reader of m^6^A RNA Modification: YTHDF1 in Merkel Cell Carcinoma. Cancers (Basel). 2020; 12:202. 10.3390/cancers12010202 31947544 PMC7016651

[50] Niu Y, Lin Z, Wan A, Chen H, Liang H, Sun L, Wang Y, Li X, Xiong XF, Wei B, Wu X, Wan G. RNA N6-methyladenosine demethylase FTO promotes breast tumor progression through inhibiting BNIP3. Mol Cancer. 2019; 18:46. 10.1186/s12943-019-1004-4 30922314 PMC6437932

[51] Nishizawa Y, Konno M, Asai A, Koseki J, Kawamoto K, Miyoshi N, Takahashi H, Nishida N, Haraguchi N, Sakai D, Kudo T, Hata T, Matsuda C, et al. Oncogene c-Myc promotes epitranscriptome m^6^A reader YTHDF1 expression in colorectal cancer. Oncotarget. 2017; 9:7476–86. 10.18632/oncotarget.23554 29484125 PMC5800917

[52] Nagaki Y, Motoyama S, Yamaguchi T, Hoshizaki M, Sato Y, Sato T, Koizumi Y, Wakita A, Kawakita Y, Imai K, Nanjo H, Watanabe H, Imai Y, et al. m^6^ A demethylase ALKBH5 promotes proliferation of esophageal squamous cell carcinoma associated with poor prognosis. Genes Cells. 2020; 25:547–61. 10.1111/gtc.12792 32449584

[53] Ma L, Lin Y, Sun SW, Xu J, Yu T, Chen WL, Zhang LH, Guo YC, Wang YW, Chen T, Wei JF, Zhu LJ. KIAA1429 is a potential prognostic marker in colorectal cancer by promoting the proliferation via downregulating WEE1 expression in an m6A-independent manner. Oncogene. 2022; 41:692–703. 10.1038/s41388-021-02066-z 34819634

[54] Lu S, Yu Z, Xiao Z, Zhang Y. Gene Signatures and Prognostic Values of m^6^A Genes in Nasopharyngeal Carcinoma. Front Oncol. 2020; 10:875. 10.3389/fonc.2020.00875 32596151 PMC7300221

[55] Liu X, Xiao M, Zhang L, Li L, Zhu G, Shen E, Lv M, Lu X, Sun Z. The m6A methyltransferase METTL14 inhibits the proliferation, migration, and invasion of gastric cancer by regulating the PI3K/AKT/mTOR signaling pathway. J Clin Lab Anal. 2021; 35:e23655. 10.1002/jcla.23655 33314339 PMC7957981

[56] Liu L, Wu Y, Li Q, Liang J, He Q, Zhao L, Chen J, Cheng M, Huang Z, Ren H, Chen J, Peng L, Gao F, et al. METTL3 Promotes Tumorigenesis and Metastasis through BMI1 m^6^A Methylation in Oral Squamous Cell Carcinoma. Mol Ther. 2020; 28:2177–90. 10.1016/j.ymthe.2020.06.024 32621798 PMC7544972

[57] Lin S, Zhu Y, Ji C, Yu W, Zhang C, Tan L, Long M, Luo D, Peng X. METTL3-Induced miR-222-3p Upregulation Inhibits STK4 and Promotes the Malignant Behaviors of Thyroid Carcinoma Cells. J Clin Endocrinol Metab. 2022; 107:474–90. 10.1210/clinem/dgab480 34562008

[58] Li Y, Zheng D, Wang F, Xu Y, Yu H, Zhang H. Expression of Demethylase Genes, FTO and ALKBH1, Is Associated with Prognosis of Gastric Cancer. Dig Dis Sci. 2019; 64:1503–13. 10.1007/s10620-018-5452-2 30637548 PMC6522448

[59] Li X, Tang J, Huang W, Wang F, Li P, Qin C, Qin Z, Zou Q, Wei J, Hua L, Yang H, Wang Z. The M6A methyltransferase METTL3: acting as a tumor suppressor in renal cell carcinoma. Oncotarget. 2017; 8:96103–16. 10.18632/oncotarget.21726 29221190 PMC5707084

[60] Li T, Hu PS, Zuo Z, Lin JF, Li X, Wu QN, Chen ZH, Zeng ZL, Wang F, Zheng J, Chen D, Li B, Kang TB, et al. METTL3 facilitates tumor progression via an m^6^A-IGF2BP2-dependent mechanism in colorectal carcinoma. Mol Cancer. 2019; 18:112. 10.1186/s12943-019-1038-7 31230592 PMC6589893

[61] Li J, Rao B, Yang J, Liu L, Huang M, Liu X, Cui G, Li C, Han Q, Yang H, Cui X, Sun R. Dysregulated m6A-Related Regulators Are Associated With Tumor Metastasis and Poor Prognosis in Osteosarcoma. Front Oncol. 2020; 10:769. 10.3389/fonc.2020.00769 32582536 PMC7280491

[62] Huang H, Li L, Chen S, Lü W, Hu J. Expression of demethylase ALKBH5 in lung adenocarcinoma and its relationship with cell proliferation. Tumor. 2018; 38:572–80. 10.3781/j.issn.1000-7431.2018.33.009

[63] Hua W, Zhao Y, Jin X, Yu D, He J, Xie D, Duan P. METTL3 promotes ovarian carcinoma growth and invasion through the regulation of AXL translation and epithelial to mesenchymal transition. Gynecol Oncol. 2018; 151:356–65. 10.1016/j.ygyno.2018.09.015 30249526

[64] Han J, Wang JZ, Yang X, Yu H, Zhou R, Lu HC, Yuan WB, Lu JC, Zhou ZJ, Lu Q, Wei JF, Yang H. METTL3 promote tumor proliferation of bladder cancer by accelerating pri-miR221/222 maturation in m6A-dependent manner. Mol Cancer. 2019; 18:110. 10.1186/s12943-019-1036-9 31228940 PMC6588935

[65] Guo YQ, Wang Q, Wang JG, Gu YJ, Song PP, Wang SY, Qian XY, Gao X. METTL3 modulates m6A modification of CDC25B and promotes head and neck squamous cell carcinoma malignant progression. Exp Hematol Oncol. 2022; 11:14. 10.1186/s40164-022-00256-3 35287752 PMC8919647

[66] Guo X, Li K, Jiang W, Hu Y, Xiao W, Huang Y, Feng Y, Pan Q, Wan R. RNA demethylase ALKBH5 prevents pancreatic cancer progression by posttranscriptional activation of PER1 in an m6A-YTHDF2-dependent manner. Mol Cancer. 2020; 19:91. 10.1186/s12943-020-01158-w 32429928 PMC7236181

[67] Du M, Peng Y, Li Y, Sun W, Zhu H, Wu J, Zong D, Wu L, He X. MYC-activated RNA N6-methyladenosine reader IGF2BP3 promotes cell proliferation and metastasis in nasopharyngeal carcinoma. Cell Death Discov. 2022; 8:53. 10.1038/s41420-022-00844-6 35136045 PMC8826370

[68] Deng R, Cheng Y, Ye S, Zhang J, Huang R, Li P, Liu H, Deng Q, Wu X, Lan P, Deng Y. m^6^A methyltransferase METTL3 suppresses colorectal cancer proliferation and migration through p38/ERK pathways. Onco Targets Ther. 2019; 12:4391–402. 10.2147/OTT.S201052 31239708 PMC6556107

[69] Chen X, Xu M, Xu X, Zeng K, Liu X, Sun L, Pan B, He B, Pan Y, Sun H, Xia X, Wang S. RETRACTED: METTL14 Suppresses CRC Progression via Regulating N6-Methyladenosine-Dependent Primary miR-375 Processing. Mol Ther. 2020; 28:599–612. 10.1016/j.ymthe.2019.11.016. Retraction in: Mol Ther. 2022; 30:2640. 10.1016/j.ymthe.2019.11.016 31839484 PMC7001002

[70] Chen HD, Li F, Chen S, Zhong ZH, Gao PF, Gao WZ. METTL3-mediated N6-methyladenosine modification of DUSP5 mRNA promotes gallbladder-cancer progression. Cancer Gene Ther. 2022; 29:1012–20. 10.1038/s41417-021-00406-5 34799724

[71] Chen XY, Zhang J, Zhu JS. The role of m^6^A RNA methylation in human cancer. Mol Cancer. 2019; 18:103. 10.1186/s12943-019-1033-z 31142332 PMC6540575

[72] Liu ZX, Li LM, Sun HL, Liu SM. Link Between m6A Modification and Cancers. Front Bioeng Biotechnol. 2018; 6:89. 10.3389/fbioe.2018.00089 30062093 PMC6055048

[73] Liu T, Yang S, Sui J, Xu SY, Cheng YP, Shen B, Zhang Y, Zhang XM, Yin LH, Pu YP, Liang GY. Dysregulated N6-methyladenosine methylation writer METTL3 contributes to the proliferation and migration of gastric cancer. J Cell Physiol. 2020; 235:548–62. 10.1002/jcp.28994 31232471

[74] Peng W, Li J, Chen R, Gu Q, Yang P, Qian W, Ji D, Wang Q, Zhang Z, Tang J, Sun Y. Upregulated METTL3 promotes metastasis of colorectal Cancer via miR-1246/SPRED2/MAPK signaling pathway. J Exp Clin Cancer Res. 2019; 38:393. 10.1186/s13046-019-1408-4 31492150 PMC6729001

[75] Liu J, Sun G, Pan S, Qin M, Ouyang R, Li Z, Huang J. The Cancer Genome Atlas (TCGA) based m^6^A methylation-related genes predict prognosis in hepatocellular carcinoma. Bioengineered. 2020; 11:759–68. 10.1080/21655979.2020.1787764 32631107 PMC8291839

[76] Yuan Y, Du Y, Wang L, Liu X. The M6A methyltransferase METTL3 promotes the development and progression of prostate carcinoma via mediating MYC methylation. J Cancer. 2020; 11:3588–95. 10.7150/jca.42338 32284755 PMC7150444

[77] Visvanathan A, Patil V, Arora A, Hegde AS, Arivazhagan A, Santosh V, Somasundaram K. Essential role of METTL3-mediated m^6^A modification in glioma stem-like cells maintenance and radioresistance. Oncogene. 2018; 37:522–33. 10.1038/onc.2017.351 28991227

[78] Yao Y, Bi Z, Wu R, Zhao Y, Liu Y, Liu Q, Wang Y, Wang X. METTL3 inhibits BMSC adipogenic differentiation by targeting the JAK1/STAT5/C/EBPβ pathway *via* an m^6^A-YTHDF2-dependent manner. FASEB J. 2019; 33:7529–44. 10.1096/fj.201802644R 30865855

[79] Zhang C, Zhang M, Ge S, Huang W, Lin X, Gao J, Gong J, Shen L. Reduced m6A modification predicts malignant phenotypes and augmented Wnt/PI3K-Akt signaling in gastric cancer. Cancer Med. 2019; 8:4766–81. 10.1002/cam4.2360 31243897 PMC6712480

[80] Bi X, Lv X, Liu D, Guo H, Yao G, Wang L, Liang X, Yang Y. METTL3-mediated maturation of miR-126-5p promotes ovarian cancer progression via PTEN-mediated PI3K/Akt/mTOR pathway. Cancer Gene Ther. 2021; 28:335–49. 10.1038/s41417-020-00222-3 32939058

[81] Cui X, Wang Z, Li J, Zhu J, Ren Z, Zhang D, Zhao W, Fan Y, Zhang D, Sun R. Cross talk between RNA N6-methyladenosine methyltransferase-like 3 and miR-186 regulates hepatoblastoma progression through Wnt/β-catenin signalling pathway. Cell Prolif. 2020; 53:e12768. 10.1111/cpr.12768 31967701 PMC7106953

[82] Zhang BH, Yan LN, Yang JY. Pending role of METTL14 in liver cancer. Hepatobiliary Surg Nutr. 2019; 8:669–70. 10.21037/hbsn.2019.10.16 31930004 PMC6943010

[83] Chen X, Xu M, Xu X, Zeng K, Liu X, Pan B, Li C, Sun L, Qin J, Xu T, He B, Pan Y, Sun H, Wang S. METTL14-mediated N6-methyladenosine modification of SOX4 mRNA inhibits tumor metastasis in colorectal cancer. Mol Cancer. 2020; 19:106. 10.1186/s12943-020-01220-7 32552762 PMC7298962

[84] Li Z, Li F, Peng Y, Fang J, Zhou J. Identification of three m6A-related mRNAs signature and risk score for the prognostication of hepatocellular carcinoma. Cancer Med. 2020; 9:1877–89. 10.1002/cam4.2833 31943856 PMC7050095

[85] Panneerdoss S, Eedunuri VK, Yadav P, Timilsina S, Rajamanickam S, Viswanadhapalli S, Abdelfattah N, Onyeagucha BC, Cui X, Lai Z, Mohammad TA, Gupta YK, Huang TH, et al. Cross-talk among writers, readers, and erasers of m^6^A regulates cancer growth and progression. Sci Adv. 2018; 4:eaar8263. 10.1126/sciadv.aar8263 30306128 PMC6170038

[86] You Y, Wen D, Zeng L, Lu J, Xiao X, Chen Y, Song H, Liu Z. ALKBH5/MAP3K8 axis regulates PD-L1+ macrophage infiltration and promotes hepatocellular carcinoma progression. Int J Biol Sci. 2022; 18:5001–18. 10.7150/ijbs.70149 35982895 PMC9379398

[87] Sun T, Wu R, Ming L. The role of m6A RNA methylation in cancer. Biomed Pharmacother. 2019; 112:108613. 10.1016/j.biopha.2019.108613 30784918

