# Supplementary material for: Prognostic and clinicopathological value of m6A regulators in human cancers: a meta-analysis
Source: Aging (Albany NY). 2022 Nov 7;14(21):8818–38. doi: 10.18632/aging.204371 (PMC9699754; doi:10.18632/aging.204371)

**Supplementary Table 1. Search history.**

Pubmed

| Query | Results |
| --- | --- |
| (((Carcinoma[Title/Abstract]) OR (((((((((((((((((((("Neoplasms"[Mesh]) OR (Tumor[Title/Abstract])) OR (Neoplasm[Title/Abstract])) OR (Tumors[Title/Abstract])) OR (Neoplasia[Title/Abstract])) OR (Neoplasias[Title/Abstract])) OR (Cancer[Title/Abstract])) OR (Cancers[Title/Abstract])) OR (Malignant Neoplasm[Title/Abstract])) OR (Malignancy[Title/Abstract])) OR (Malignancies[Title/Abstract])) OR (Malignant Neoplasms[Title/Abstract])) OR (Neoplasm, Malignant[Title/Abstract])) OR (Neoplasms, Malignant[Title/Abstract])) OR (Benign Neoplasms[Title/Abstract])) OR (Benign Neoplasm[Title/Abstract])) OR (Neoplasms, Benign[Title/Abstract])) OR (Neoplasm, Benign[Title/Abstract]))))) AND ((("N-methyladenosine" [Supplementary Concept]) OR (((((N(6)-methyladenosine[Title/Abstract]) OR (N(6)mAdo[Title/Abstract])) OR (N6-methyladenosine[Title/Abstract])) OR (N6-methyladenosine (m6A[Title/Abstract]))) OR (6-methyladenosine[Title/Abstract]))))) AND ((("Survival"[Mesh]) OR (("Prognosis"[Mesh]) OR ((((((Prognoses[Title/Abstract]) OR (Prognostic Factors[Title/Abstract])) OR (Prognostic Factor[Title/Abstract])) OR (Factor, Prognostic[Title/Abstract])) OR (Factors, Prognostic[Title/Abstract])) OR (Prognostic[Title/Abstract])))) OR (outcome[Title/Abstract])) | 544 |

Cochrance


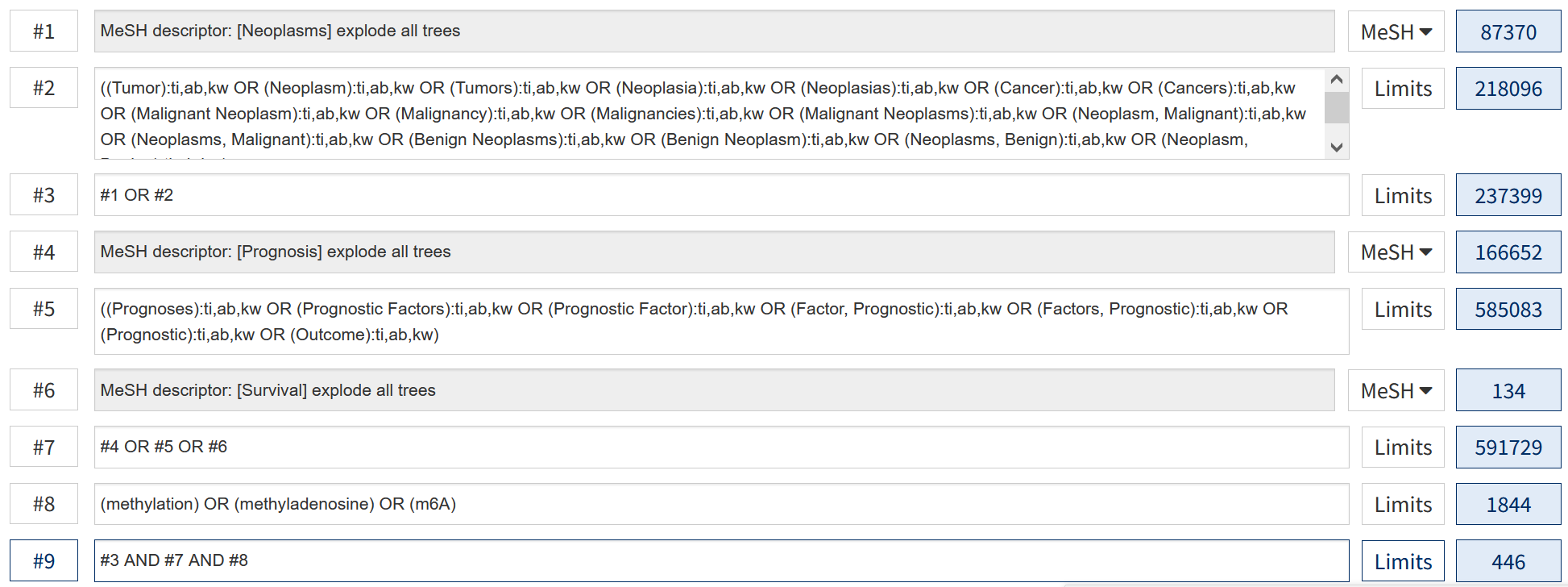


Embase Session Results

| No. | Query | Results |
| --- | --- | --- |
| #10 | #7 AND #8 AND #9 | **1071** |
| #9 | #3 OR #6 | **3619567** |
| #8 | #2 OR #5 | **4361** |
| #7 | #1 OR #4 | **6457812** |
| #6 | 'prognoses':ab,ti OR 'prognostic factors':ab,ti OR 'prognostic factor':ab,ti OR 'factor, prognostic':ab,ti OR 'factors, prognostic':ab,ti OR 'prognostic':ab,ti OR 'outcome':ab,ti OR 'survival':ab,ti | **3247717** |
| #5 | 'n(6)-methyladenosine':ab,ti OR 'n(6)mado':ab,ti OR 'n6-methyladenosine':ab,ti OR 'n6-methyladenosine (m6a)':ab,ti OR 'm6a':ab,ti OR '6-methyladenosine':ab,ti | **3879** |
| #4 | 'tumor':ab,ti OR 'neoplasm':ab,ti OR 'tumors':ab,ti OR 'neoplasia':ab,ti OR 'neoplasias':ab,ti OR 'cancer':ab,ti OR 'cancers':ab,ti OR 'malignant neoplasm':ab,ti OR 'malignancy':ab,ti OR 'malignancies':ab,ti OR 'malignant neoplasms':ab,ti OR 'neoplasm, malignant':ab,ti OR 'neoplasms, malignant':ab,ti OR 'benign neoplasms':ab,ti OR 'benign neoplasm':ab,ti OR 'neoplasms, benign':ab,ti OR 'neoplasm, benign':ab,ti OR 'carcinoma':ab,ti | **4613109** |
| #3 | 'prognosis'/exp | **853204** |
| #2 | 'n-methyladenosine'/exp | **2813** |
| #1 | 'neoplasms'/exp | **5621825** |

Web of sci


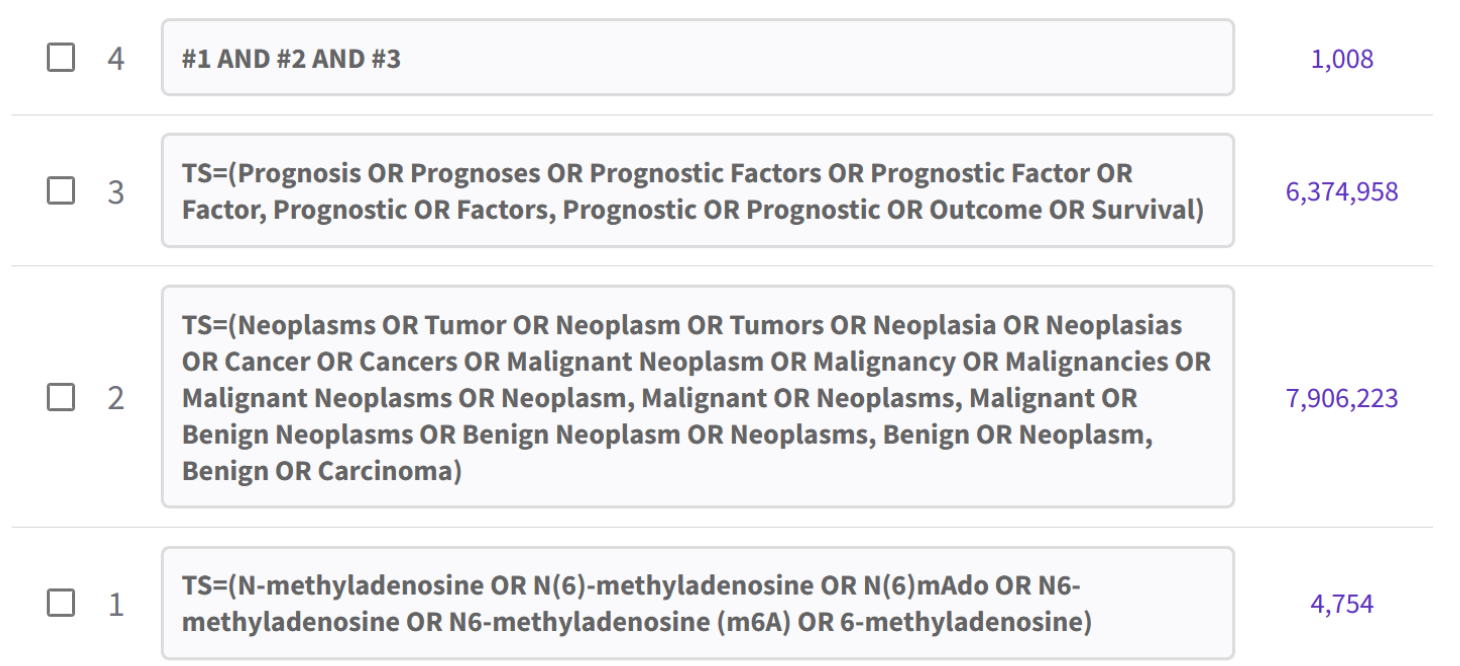

Supplement: Supplementary Table 1 [file aging-14-204371-s001.docx]
